# Non-order–disorder allotwinning of the rhenium pincer complex *cis*-Re[(PNP^CH2^-*i*Pr)(CO)_2_Cl]

**DOI:** 10.1107/S205252061701006X

**Published:** 2017-09-27

**Authors:** Mathias Glatz, Berthold Stöger, Karl Kirchner

**Affiliations:** a Institute of Applied Synthethic Chemistry, TU Wien, Getreidemarkt 9, 1060 Vienna, Austria; b X-ray Centre, TU Wien, Getreidemarkt 9, 1060 Vienna, Austria

**Keywords:** polytypism, allotwinning

## Abstract

Crystals of *cis*-Re[(PNP^CH2^-*i*Pr)(CO)_2_Cl] (1) are made up of two geometrically non-equivalent polytypes with respective symmetries of *P*2_1_/*c* and *I*2/*a*. The structures were determined in a concurrent refinement, taking into account overlap of diffraction spots. The polytypes are composed of layers with *p*_*x*_12_1_/*c*1 symmetry and are of the non-order–disorder (OD) type (the layer interfaces are non-equivalent). Whereas the molecules of (1) differ in both polytypes, the Re atoms are located at nearly identical positions.

## Abstract 2

Crystals of *cis*-Re[(PNP^CH2^-*i*Pr)(CO)_2_Cl] are made up of two distinct non-order–disorder polytypes.

## 1. Introduction   

Polytypes are modular structures (Ferraris *et al.*, 2008 ▸) that are composed of equivalent layers (or more generally rods or blocks) arranged into non-equivalent stackings. Polytypes, which are ubiquitous in all classes of materials, can crystallize with different degrees of order, ranging from perfectly ordered to purely random stackings. When crystallizing with only few stacking faults, polytypes often form twins, which are made up of macroscopic equivalent domains with different orientations (Hahn & Klapper, 2006 ▸).

A cognate phenomenon is allotwinning (Nespolo *et al.*, 1999 ▸). These edifices are made up of crystalline domains of *different* polytypes. Apparently, in allotwins the crystallization conditions vary in such a way that only one of two or more polytypes is formed at a time. An alternative formation mechanism that has been proposed is oriented attachment (Nespolo & Ferraris, 2004 ▸), where polytypes of different kinds form at different places and attach post-nucleation in a systematic manner.

Intuitively, both formation mechanisms appear unlikely and indeed examples of allotwins which have been structurally properly characterized are rare. In contrast, our experience with single-crystal diffraction of inorganic, organic and coordination compounds suggests an orders-of-magnitude higher frequency of allotwins than would be inferred from their reported number.

**Figure 1:**
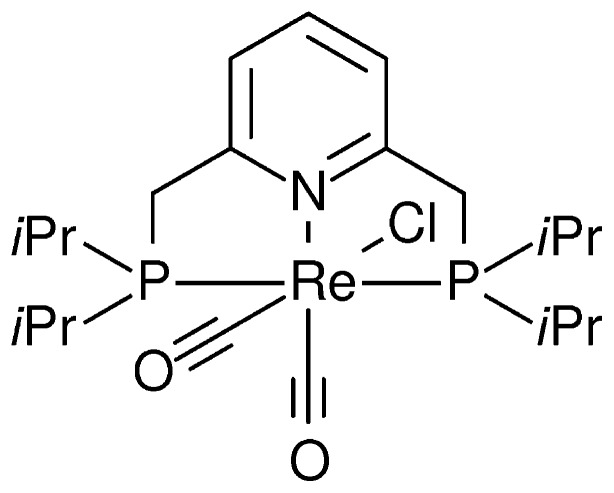
Scheme of complex (1), *cis*-Re[(PNP^CH2^-*i*Pr)(CO)_2_Cl].

One reason for the under-reporting is certainly the missing support in the common crystallographic software packages. But the biggest hurdle might actually be a failure of recognizing allotwinning, owing to a lack of awareness of the phenomenon. In this communication both points are addressed by giving a detailed account of the structure refinement of an allotwinned crystal of the Re^I^ complex *cis*-Re[(PNP^CH2^-*i*Pr)(CO)_2_Cl] [(1), Fig. 1 ▸]. It is shown that, once the nature of the diffraction pattern is understood and proper intensity data are derived, structure solution and refinement can be surprisingly trouble-free. The ^*t*^Bu analogue of (1), *cis*-Re[(PNP^CH2^-*t*Bu)(CO)_2_Cl], has been described previously (Vogt *et al.*, 2013 ▸) and does not feature polytypism.

Data reduction is a crucial step in the characterization of allotwins. In the simplest case, the polytypes share a common sublattice (used here in the sense of a common subset of translation vectors) and the set of overlapping reflections is well defined. The reflections of all polytypes can then be integrated concurrently using a common superlattice in reciprocal space. Unfortunately, this superlattice is often rather dense, leading to a large number of virtual overlaps of non-existing diffraction spots, and in consequence to suboptimal intensity evaluation.

In the general case, the matrix describing the lattice relationship is non-rational and reflections are partially overlapping. The common strategy (also previously used for classical twinning) then has been to integrate the data of the individuals separately and determine overlaps by heuristics (*i.e.* considering all the reflections separated by less than a threshold value as overlapped). For classical twinning, the concurrent integration of multiple domains with overlap information has become a standard. The advantage is that the integration software is aware of the reflection-mask shape and therefore can precisely determine the amount of overlap. We have recently applied such an approach to a multi-domain crystal (Stöger *et al.*, 2015 ▸).

Structure refinements of allotwins can likewise follow two major strategies. Either the models of the individual polytype are refined separately against the non-overlapping reflections or in a concurrent refinement taking into account reflection overlaps. Since allotwins are by definition oriented systematically, reflection overlap is likewise systematic and therefore the latter approach is preferable, even though only few refinement packages support such refinements.

Here, we want to advocate an integration with overlap information followed by a concurrent refinement against the full data set. Such a scheme represents the most controlled and satisfying approach, avoiding heuristics as much as possible.

## 2. Experimental   

### 2.1. Synthesis and crystal growth   

The PNP^CH2^-*i*Pr ligand was synthesized according to literature procedures (Leung *et al.*, 2003 ▸). PNP^CH2^-*i*Pr (136 mg, 0.4 mmol) and Re(CO)_5_Cl (144 mg, 0.4 mmol) were refluxed in dioxane (10 ml) for 72 h. The suspension was evaporated to dryness, taken up in dry acetone and filtered over celite. The solvent was removed under reduced pressure, the pale yellow residue washed with *n*-pentane (15 ml) and dried under reduced pressure. Yellow crystals of (1) were grown by vapor diffusion of *n*-pentane into a CH_2_Cl_2_ solution of the crude product. Colorless crystals of Re[(PNP^CH2^-*i*Pr)(CO)_3_]·Cl were obtained as a side product. Anal.: calc. for C_21_H_35_ClNO_2_P_2_Re (617.12): C 40.87, H 5.72, N 2.27; found: C 40.90, H 5.77, N 2.27%. ^1^H NMR (600 MHz, δ, CD_2_Cl_2_, 20°C) 7.55 (t, 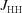 = 7.7 Hz, ^1^H, py^4^), 7.25 (d, 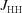 = 7.7 Hz, 2H, py^3,5^), 3.88 (m, 2H, CH_2_), 2.48 (m, 2H, CH_2_), 2.72 (m, 2H, CH), 2.40 (m, 2H, CH), 1.26–1.20 (m, 18H, CH_3_), 1.09 (dd, 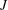 = 15.1, 7.3 Hz, 6H, CH_3_). ^13^C{^1^H} NMR (151 MHz, δ, CD_2_Cl_2_, 20°C) 208.9 (m, CO), 199.2 (vt, 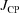 = 8.2 Hz, CO), 164.4 (vt, 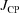 = 4.6 Hz, py^2,6^), 137.4 (s, py^4^), 120.5 (vt, 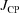 = 4.4 Hz, py^3,5^), 42.9 (vt, 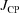 = 11.2 Hz, CH_2_), 26.9 (vt, 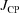 = 13.5 Hz, CH), 24.3 (vt, 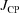 = 11.7 Hz, CH), 19.8 (vt, 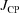 = 1.8 Hz, CH_3_), 19.7 (vt, 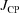 = 1.5 Hz, CH_3_), 19.3 (s, CH_3_), 17.7 (s, CH_3_). ^13^P{^1^H} NMR (101 MHz, δ, CD_2_Cl_2_, 20°C) 52.4 (2P). IR (ATR, cm^−1^): 1900 (νCO), 1806 (νCO).

### 2.2. Data collection   

The yellow blocks of (1) were optically homogeneous, but cleaved into numerous small platelets on cutting with a razor blade. Generally, diffraction quality was mediocre (arcing, splitting of reflections), being worse for larger crystals. Therefore, intensity data of a tiny block as-grown was collected at 200 K in a dry stream of nitrogen on a Bruker KAPPA APEX II diffractometer system using graphite monochromated Mo 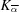 radiation and fine sliced ω- and φ-scans. The whole reciprocal sphere up to 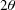 = 60° was collected. Data collection and refinement details are summarized in Tables 1 ▸ and 2 ▸.

### 2.3. Cell determination and integration   

Depending on the chosen tolerances, automatic unit cell determination with the *Apex*3 (Bruker, 2014 ▸) software yielded different (non-equivalent) orientation matrices, none of which was able to explain the majority of the diffraction spots. All the proposed cells were metrically monoclinic and shared a common **b** ^*^ basis vector. Indeed, as observed in the *RLATT* module (Bruker, 2014 ▸), virtually all reflections were located in planes normal to **b** ^*^. The few remaining reflections between these planes were attributed to negligible admixtures and culled for ease of further processing.

**Figure 2:**
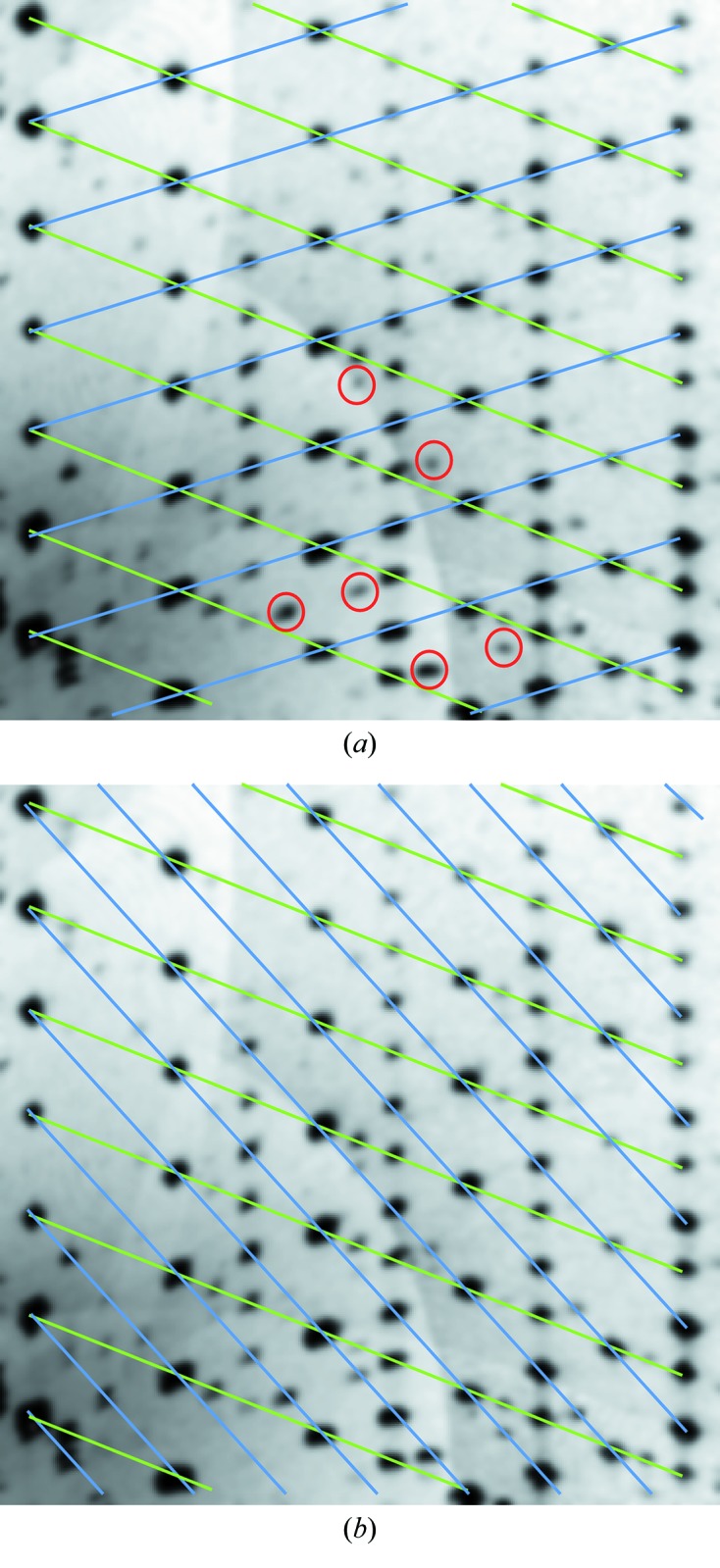
Two indexing attempts of the (1) crystal under investigation shown at the *h*2*l* plane of reciprocal space reconstructed from CCD images. Green lines: *mP*. Blue lines: *mC*. Examples of faint superstructure reflections which led to the reindexing (*b*) are marked in (*a*) by red circles.

A view along **b** ^*^ revealed two kinds of reciprocal lattice rows, which span different lattices as indicated in the reconstructed lattice plane in Fig. 2(*a*) ▸. These rows were intuitively interpreted as a sign of twinning with a twin index > 1 and therefore the reflections were separated and the orientation matrices determined individually. Two satisfying lattices were thus obtained, albeit belonging to different Bravais classes (*mP* and *mC*).

After proceeding as described in the following section, no chemically reasonable structure refinement was possible for the *mC* domain. In all cases, even with *C*1 symmetry, a virtual overlap of (1) complexes in two orientations was obtained, suggesting an erroneous lattice. Therefore, the diffraction pattern was re-evaluated and indeed weak reflections that are potential superstructure reflections of the *mC* domain were identified [red circles in Fig. 2(*a*) ▸]. Thus, the lattice of the *mC* domain was reindexed as shown in Fig. 2(*b*) ▸. The resulting lattice still was of the *mC* kind, but featured a doubled cell volume. It is thus shown that presumably negligible faint reflections can be crucial.

Owing to software limitations (lack of support of concurrent integration with different Bravais lattices) both domains were integrated in the primitive reduced settings without restrictions on the cell parameters and with overlap information (HKLF5 style format) using *SAINT-Plus* (Bruker, 2014 ▸). In such an integration, overlapping reflections are reduced to a single intensity datum associated with two *hkl* indices. The *hkl* indices were later retransformed into the proper monoclinic settings.

To achieve a smooth integration without an excess of discarded reflections, the integration parameters had to be optimized. Notably, the allowed common volume of non-overlapping reflections had to be increased from the default 4% to 15%. A correction for absorption effects was then applied using the multi-scan approach implemented in *TWINABS* (Bruker, 2014 ▸).

### 2.4. Structure solution and refinement   

In a first step, the non-overlapping reflections of both domains were separated and the overlapping reflections were discarded. The two independent data sets were used for structure solution using the dual-space approach implemented in *SHELXT* (Sheldrick, 2015 ▸). Both models were refined using *Jana*2006 (Petříček *et al.*, 2014 ▸), resulting in satisfactory reliability factors. The correct space groups could thus unambiguously be identified as *P*2_1_/*c* and *I*2/*a*. The reduced *I*-centered setting was used for a better comparability of both structures (shared layer lattice vectors **b** and **c**). The models were then combined to a two-phase model and the reflection data were replaced by the HKLF5 file with overlap information. The volume fraction of both domains was refined to a *P*2_1_/*c*:*I*2/*a* ratio of ∼1:4.

In the major *I*2/*a* domain, all non-H atoms were refined with anisotropic atomic displacement parameters (ADPs). In the minor *P*2_1_/*c* domain, only the heavy atoms (Re, Cl, P) and the C atoms of the methyl groups were refined with anisotropic ADPs. In both domains, the molecules of (1) were disordered with respect to the CO and Cl ligands *cis* to N. The CO:Cl occupation ratio was refined independently for both phases to ∼4:3 (*P*2_1_/*c*) and ∼3:2 (*I*2/*a*). The ADPs of the related positions were constrained to be identical. H atoms were placed at calculated positions and refined as riding on their parent C atoms.

## 3. Results and discussion   

### 3.1. Molecular structure   

**Figure 3:**
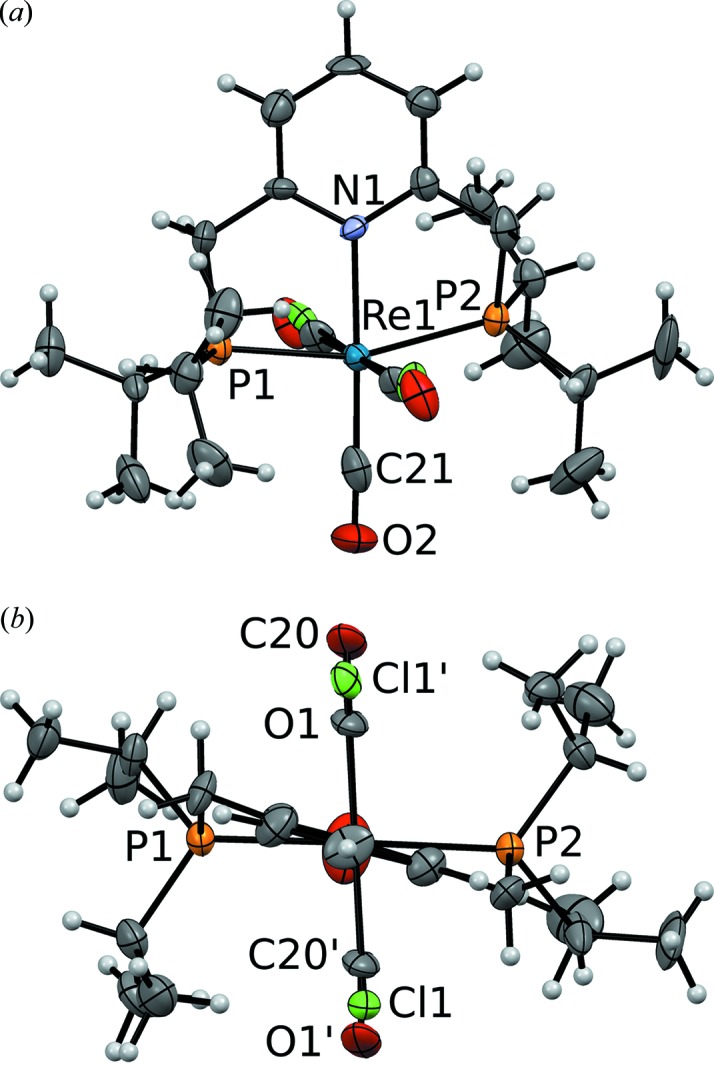
The molecular structure of (1) showing (*a*) octahedral coordination and (*b*) twofold pseudo-rotation symmetry. C (gray), N (blue), O (red), P (orange), Cl (green) and Re (dark blue) atoms are represented by ellipsoids drawn at the 50% probability levels. H atoms are represented by spheres of arbitrary radius. Atom names with a prime character designate the minor positions of the Cl and CO groups. Data taken from the *I*2/*a* model.

Complex (1) adopts an octahedral coordination (Fig. 3 ▸), which is characteristic of this class of compounds (Vogt *et al.*, 2013 ▸) and will not be expanded upon. Including the disordered Cl and CO ligands, the complex has twofold rotational pseudo-symmetry [Fig. 3(*b*) ▸] with the rotation axis passing through the pyridine ring, the Re atom and the CO ligand *trans* to N.

### 3.2. Polytypism   

**Figure 4:**
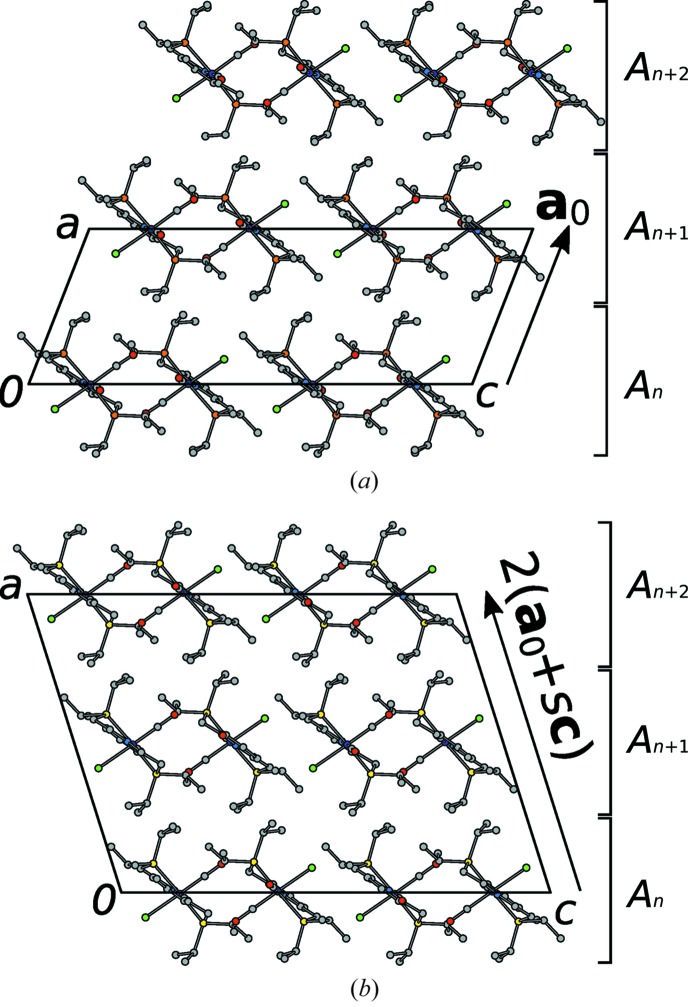
Arrangement of 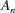 layers in the (*a*) *P*2_1_/*c* and (*b*) *I*2/*a* polytypes of (1). Atoms are represented by spheres of arbitrary radius with the color codes of Fig. 3 ▸. H atoms and the minor components of the CO/Cl disorder were omitted for clarity.

**Figure 5:**
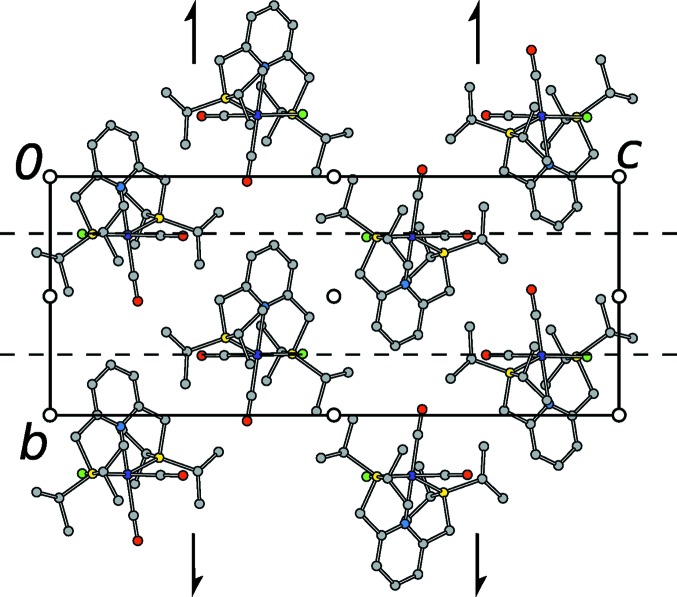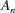 layer projected on the (100) layer plane. Atoms as in Fig. 4 ▸. A unit cell of the layer is indicated. Symmetry operations of layers and operations relating adjacent layers are represented using the usual graphic symbols (Hahn, 2006 ▸).

In both observed polytypes, the molecules of (1) are arranged in layers with 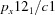 symmetry (Kopsky & Litvin, 2006 ▸), where the *x* subscript indicates a lack of translation in the [100] direction (Fig. 4 ▸). These layers will be designated as 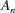, where *n* is a sequential number (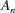 connects to 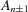, *etc*.). The rectangular layer lattice is spanned by the basis 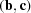. The layers are virtually equivalent in both polytypes (see §3.7). The arrangement of the molecules of (1) in an 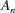 layer is shown in Fig. 5 ▸.

**Figure 6:**
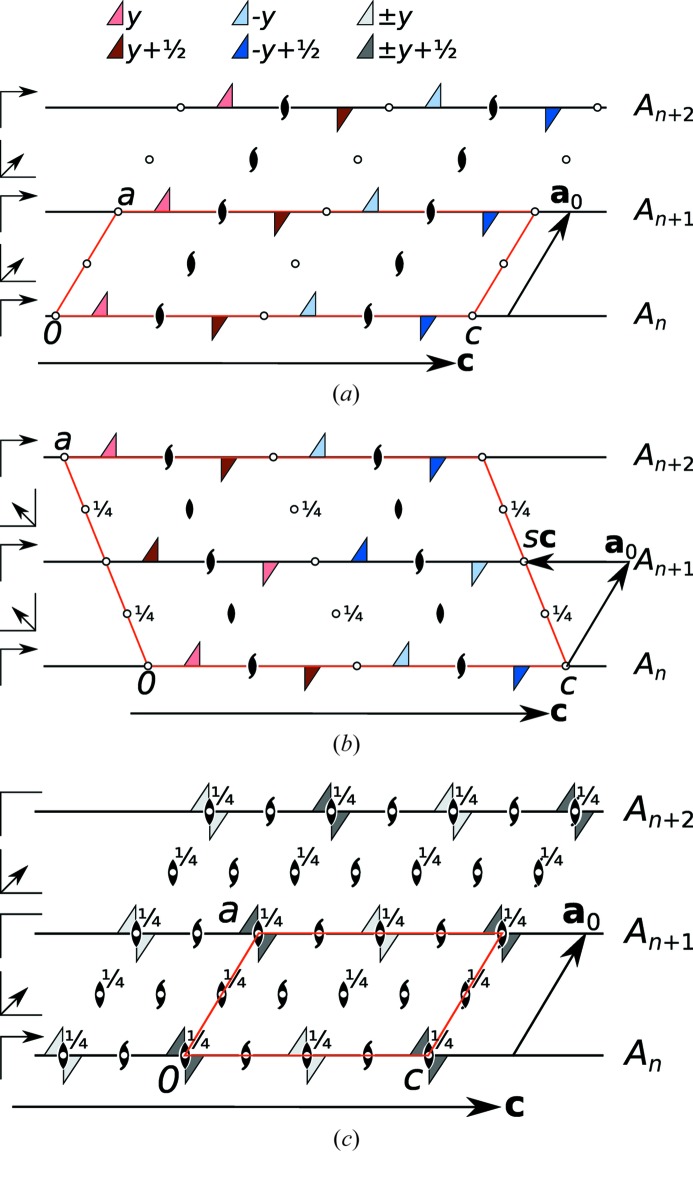
Symmetry of the 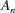 layers in (*a*) the *P*2_1_/*c* and (*b*) the *I*2/*a* polytypes as well as (*c*) the family structure of the polytype family. (1) complexes are represented by triangles which are red on one side and blue on the other side. If both orientations are realised (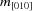 symmetry), the triangle is gray. A translation by 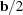 is represented by darker shading.

By definition, the polytypes differ in the stacking of the layers. In both domains adjacent layers are related by translations, though with different translation vectors. In the *P*2_1_/*c* domain, layers are related by translation with the vector 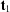 = 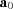, which is perpendicular to 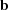. For the *I*2/*a* domain, on the other hand, adjacent layers are translationally related by 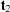 = 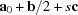. The set of operations (modulo lattice translations) relating adjacent layers are compiled in Table 3 ▸. The overall symmetries of both polytypes are schematized in Figs. 6(*a*) and 6(*b*) ▸.

The metric parameter *s* in the definition of 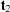 calculates from the cell parameters as 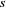 = −0.247 ≃ −1/4. Here, the translations connecting adjacent layers are expressed based on the vector 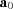, which was arbitrarily chosen to be a lattice basis vector of the *P*2_1_/*c* polytype. 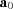 could also have been chosen based on the *I*2/*a* polytype or as being perpendicular to the layer plane. The latter would complicate further reasoning, because it introduces two metric parameters, one per polytype. For a discussion on metric parameters in polytypes, see Fichtner (1979 ▸).

**Figure 7:**
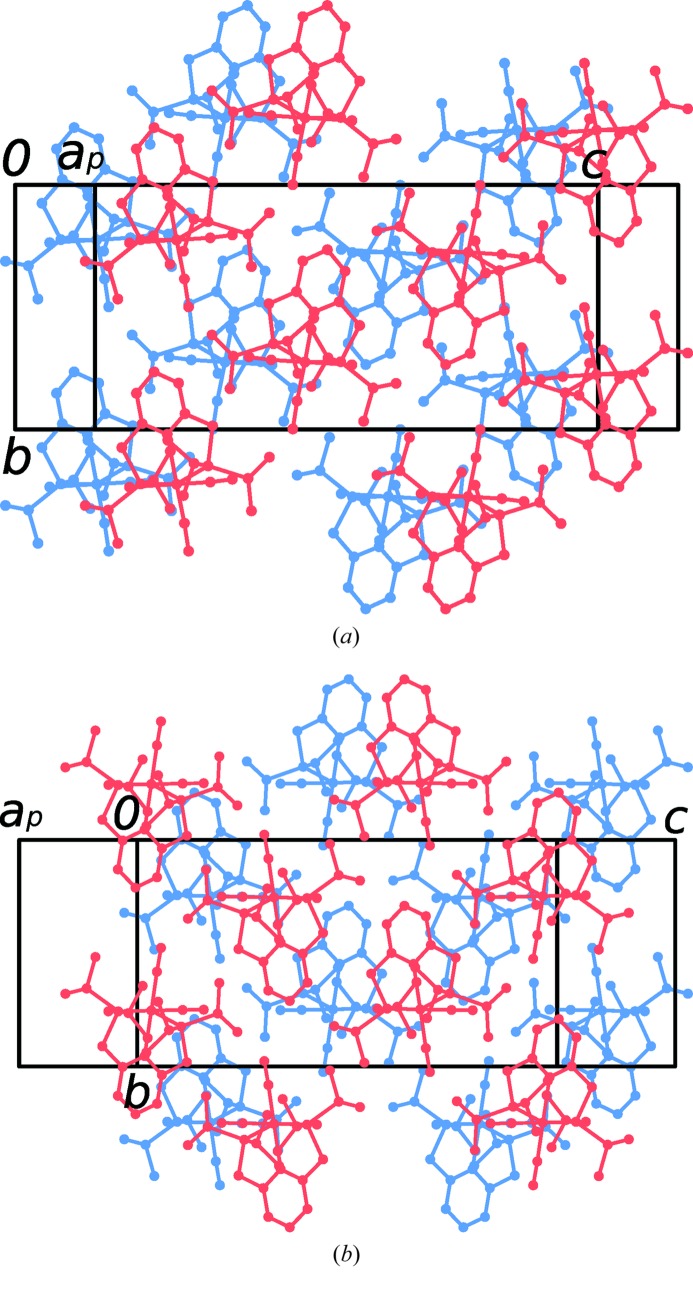
Pairs of adjacent layers in the (*a*) *P*2_1_/*c* and (*b*) *I*2/*a* polytypes of (1). Molecules in the 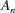 and 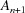 layers are blue and red, respectively.

As shown in Fig. 7 ▸, the two stacking possibilities lead to non-equivalent pairs of adjacent layers. Such polytypes are said to be of the non-order–disorder (OD) type (Ferraris *et al.*, 2008 ▸). Since every 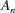 layer can contact in two ways to the adjacent 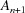 layer, the (1) complexes can in principle be arranged to an infinity of different polytypes, which all belong to the same non-OD polytype family. In the crystals of (1), two kinds of these polytypes connect *via* common layers. They can therefore be classified as *non-OD allotwins*.

### 3.3. The OD perspective   

The OD theory (Dornberger-Schiff & Grell-Niemann, 1961 ▸) was developed to explain and describe the common occurrence of polytypism in all classes of materials. Modular structures in which adjacent layers contact in only one geometrically equivalent way are said to fulfill the *vicinity condition* (VC). If the VC allows for polytypism, one speaks of *OD polytypes*. The OD theory makes a strong argument by stating that OD polytypes are locally equivalent. From the short-range interaction of atoms follows the energetic equivalence of these OD polytypes. Indeed, experience shows that most of the observed polytypes are of the OD kind.

As shown by (1), in some cases layers can also contact in non-equivalent ways. In our experience, this non-OD type of polytypism is more common in organic and coordination compounds than in classical inorganics [see, for example, Lumpi *et al.* (2015) ▸; Kader *et al.* (2017) ▸] owing to the flexibility of the side chains in these molecular compounds. Nevertheless, the symmetry formalism developed by OD theory is general and can often also be fruitfully applied to non-OD polytypes.

The symmetry of polytypes (and other modular structures) is described by partial operations (POs), which are the restrictions of motions to the subsets of Euclidean space occupied by the individual layers. Thus every PO is characterized by a motion, a source and a target layer. The composition of POs is only defined if the target of the first is the source of the second. It therefore does not form a group, but a groupoid (Brandt, 1927 ▸; Ehresmann, 1957 ▸; Ito & Sadanaga, 1976 ▸).

Inside each of the two (1) polytypes, all pairs of layers are equivalent. Thus, they fulfill VC of OD structures. But, since the *c* glide planes of adjacent layers overlap in both cases, there is only one way to achieve these particular pairs of layers [for a discussion on stacking possibilities in OD structures see Ďurovič (1997 ▸)]. In terms of OD theory, both polytypes are *fully ordered*. In other words, they belong to OD families with only one member.

OD groupoid families classify groupoids of OD polytypes in analogy to space group types for space groups (Dornberger-Schiff & Grell-Niemann, 1961 ▸; Fichtner, 1977 ▸). OD groupoids belonging to the same OD groupoid family are built according to the same symmetry principle but may differ in metrics (of layer lattices and translational components of operations relating adjacent layers) and concrete stacking arrangements. Since the linear parts of the POs relating adjacent layers are equivalent in both polytypes (Table 3 ▸), the groupoids of both polytypes belong to the same OD groupoid family.

In summary, both polytypes of (1) belong to a non-OD family of polytypes. Interpreted as OD structures, they belong to different single-member OD families, which are associated with the same OD groupoid family.

### 3.4. MDO polytypes   

For any polytype family (OD or non-OD), there is a finite set of particularly simple polytypes which are said to be of a maximum degree of order (MDO). MDO polytypes cannot be decomposed into simpler polytypes (*i.e.* into polytypes that are made up of only a selection of pairs, triples and generally *n*-tuples). Experience shows that the vast majority of ordered polytypes are of the MDO kind. Since the polytypes in the family of (1) contain different pairs of layers, the MDO polytypes are those that are made up of only one kind of pairs. These are precisely the two observed polytypes of (1).

### 3.5. Family structure   

The family structure of a polytype family is the fictitious structure that is obtained if all stacking possibilities are realised to the same degree. Determination of the family structure is often a crucial step in categorizing polytype families and the interpretation of diffraction patterns.

The symmetry of the family structure contains the symmetries of all polytypes as subgroups. It depends on the metric parameter *s* (see §3.2). Here, we will assume 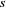 = −¼. The vector connecting the origins of both possible 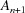 layers for a given 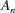 layer is 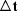 = 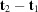 = 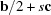 = 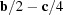. 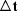 must be a translation vector of the family structure. Multiplication of 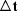 into the translation lattice of either polytype leads to a monoclinic *C*-centered (*mC*) lattice with the centered basis 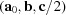 [Fig. 6(*c*) ▸].

If this lattice is applied to either of the two polytypes, the (1) complexes are an equal disorder of all four orientations observed in the 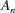 layers (site symmetry 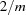). This structure is the family structure, since it contains the symmetry operations of all possible polytypes. Its overall symmetry is 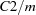 [Fig. 6(*c*) ▸].

It has to be noted that the Re atoms in the 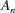 layer are located practically on the 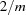 position of the family structure. Thus, the locations of the Re atoms are close to identical in all polytypes, only the ligands can adopt one out of four orientations.

### 3.6. Twinning and stacking faults   

Classical twinning is the oriented association of geometrically equivalent domains with *different orientations*. In contrast, both observed polytypes of (1) possess the same oriented point group 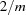. The allotwin operation (Nespolo *et al.*, 1999 ▸) is the identity, which is not a valid twin operation in classical twins.

Moreover, the 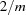 point group of both observed polytypes is precisely the point group of the polytype family (the group generated by the linear parts of all POs). Thus, in any stacking arrangement following the rules described above, both polytypes can appear in only [2/*m*:2/*m*] = 1 orientation.

A stacking fault in one polytype can only lead to domains with the same orientation, but related by a non-lattice translation. These kinds of edifices are not twins but have been designated as antiphase domains (Wondratschek & Jeitschko, 1976 ▸). In contrast to twins, such domains are hard to show and even harder to evaluate in a quantitative manner. Here, the existence of such stacking faults inside the polytypes can only be presumed owing to the generally mediocre diffraction quality.

### 3.7. Desymmetrization   

A characteristic phenomenon in polytypes is desymmetrization (Ďurovič, 1979 ▸). The most notable expression of desymmetrization in OD structures is a lowering of the actual layer symmetry compared with the idealized layer symmetry. In fully ordered structures, on the other hand, the layers typically retain their full symmetry. Indeed, in both observed polytypes of (1), the actual 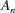 layers retain their 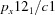 symmetry from the idealized description.

Besides a reduction in symmetry, a deviation of the geometries of the layers across polytypes can also be regarded as desymmetrization. Indeed, the determined metric parameters of the (1) polytypes differ slightly. The *b* parameter of the *P*2_1_/*c* polytype is slightly smaller than that of the *I*2/*a* polytype [10.7392 (8) *versus* 10.7708 (8) Å]. The *c* parameter shows the opposite behavior [25.629 (2) *versus* 25.599 (3) Å], resulting in essentially identical fundamental surfaces of the layer lattices (275.23 *versus* 275.72 Å^2^). The layers in the *P*2_1_/*c* polytype are marginally thicker (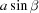 = 8.988 *versus* 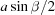 = 8.911 Å).

**Figure 8:**
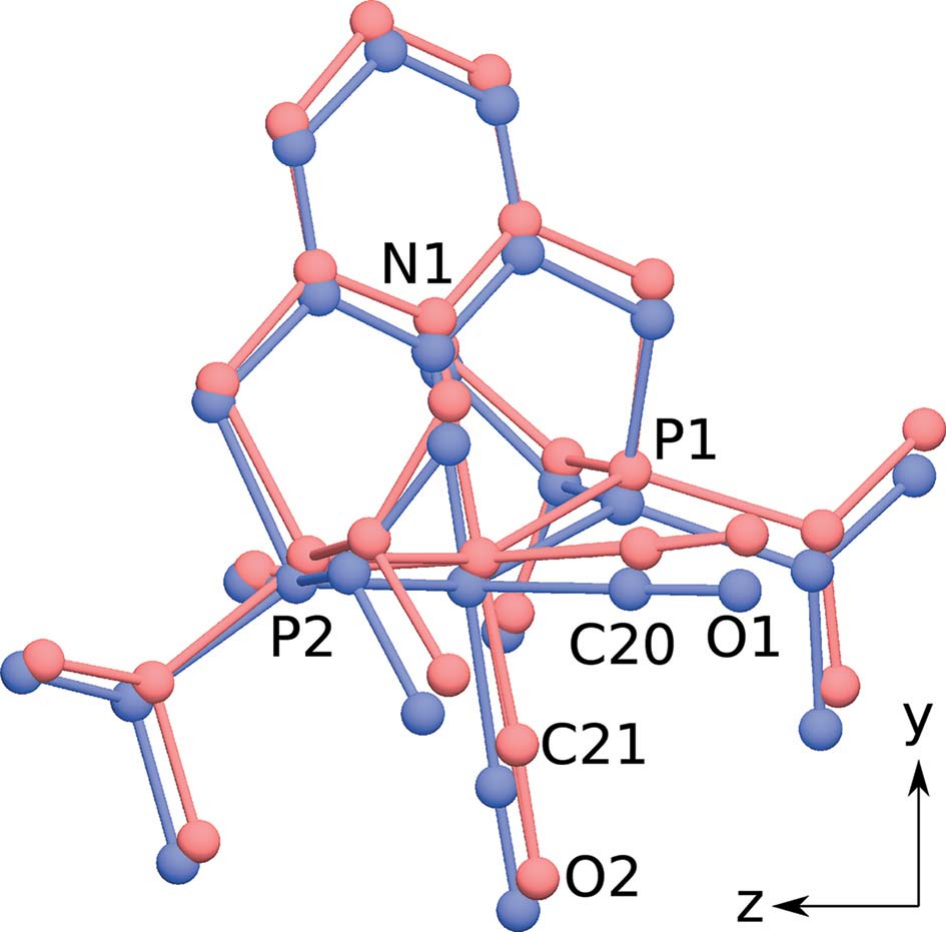
Overlay of the molecules of (1) of both polytypes projected on (100). Red: *P*2_1_/*c* polytype. Blue: *I*2/*a* polytype.

A finer evaluation of desymmetrization was obtained by transforming the coordinates of both polytypes into a Cartesian coordinate system (retaining the origin) and calculating the distances of the corresponding atoms. Neither the position nor the orientation of the complexes were optimized. An overlay of both polytypes is shown in Fig. 8 ▸ and the deviations are compiled in Table 4 ▸. The desymmetrization is substantial (up to ∼0.5 Å), which is expected since the layers are located in different environments. Nevertheless, it is clearly within the range expected for polytypes. The major contributing factor to the desymmetrization is a distinct shift of the complexes along the [010] direction. The variations of the occupancies of the disordered CO and Cl groups [72.6:27.4 (11) (∼4:3, *P*2_1_/*c*) *versus* 65.3:34.7 (∼3:2, *I*2/*a*)] can likewise be regarded as an effect of desymmetrization.

### 3.8. The layer interface   

As noted above, pairs of adjacent 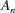 layers are non-equivalent and therefore the polytypes of (1) are of the non-OD type. Nevertheless, one has to realise that the choice of OD layers is always a matter of interpretation. By choosing non-crystallochemical layers, one might very well turn a non-OD into an OD interpretation, where all polytypes are locally equivalent. Therefore it is necessary to scrutinize the layer contact for common features and pseudo-symmetry.

**Figure 9:**
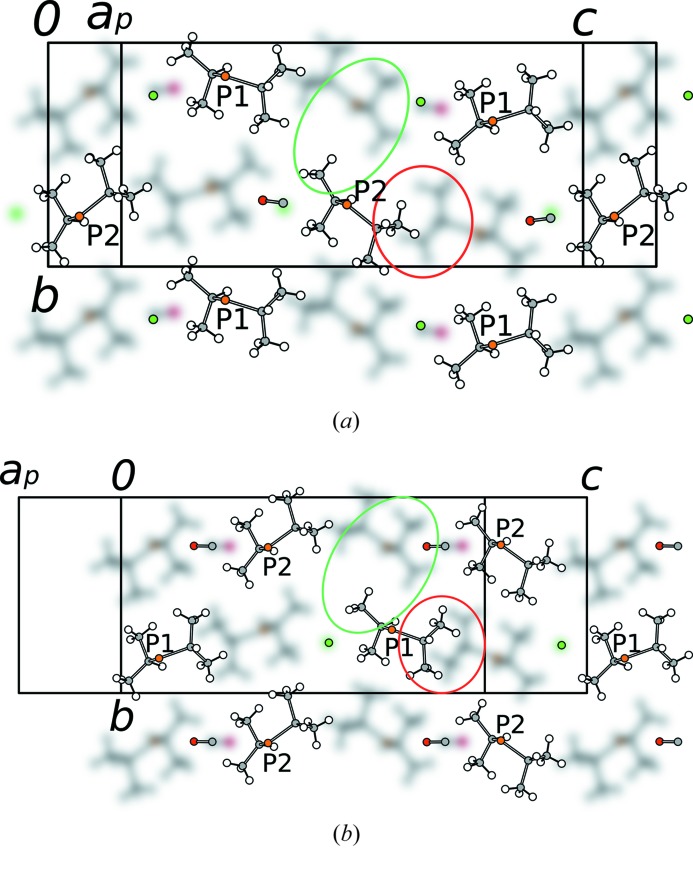
The interface between two layers in (*a*) the *P*2_1_/*c* and (*b*) the *I*2/*a* polytype of (1) projected on the layer plane (100). Only the P*i*Pr_2_ groups and the CO and Cl ligands at the interface are shown. The bottom layer is blurred. Examples of equivalent and non-equivalent inter-layer contacts are circled in green and red, respectively.

In Fig. 9 ▸ the layer contacts in both cases of 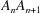 pairs are shown. Whereas at some points the contacts are similar (green ellipses), at others there are interatomic contacts not observed in the other pair (red ellipses). The polytypism can therefore be indeed considered of the non-OD type.

Abstracting from the orientations of the molecules, a common feature of both stacking arrangements is that the isopropyl groups protrude into voids in the adjacent layer. One can say that the polytypism is enabled by the fact that both the P1 and the P2 P*i*Pr_2_ groups fit into the same void (Fig. 9 ▸). In this light, the interface possesses pseudo-symmetry relating the P1 and P2 P*i*Pr_2_ groups. Nevertheless, the deviation from idealized symmetry is too pronounced for the polytypes to be considered of the OD type. A direct consequence of this structural feature is the value of the metric parameter *s* ∼ −¼ and the nearly identical positions of the Re atoms in all polytypes.

Owing to this arrangement, the CO and Cl ligands are located above either a CO or Cl ligand of the adjacent layer. In the *P*2_1_/*c* polytype the major position CO ligand in one layer is located above the major position of another CO ligand. In the *I*2/*a* polytype, on the other hand, CO mostly contacts to Cl. Thus, the CO and Cl ligands may contact to the same or different types, enabling the observed CO/Cl disorder.

### 3.9. Diffraction pattern   

**Figure 10:**
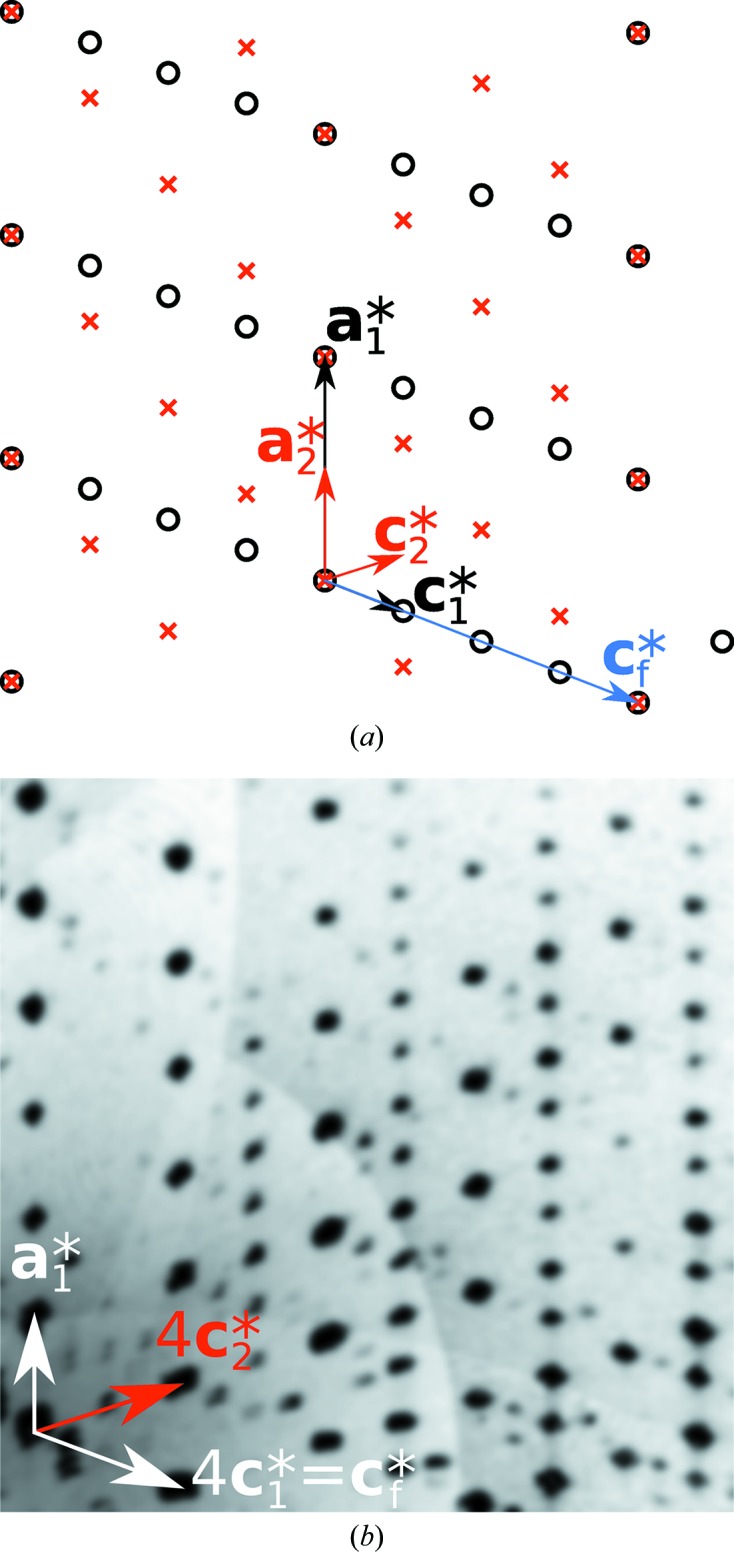
*h*2*l* plane of reciprocal space of the (1) crystal under investigation (*a*) as a scheme and (*b*) reconstructed from CCD images. In (*a*), reflections of the *P*2_1_/*c* and *I*2/*a* domains are represented by circles and crosses, respectively.

In the following discussion, *hkl* indexes will be given with respect to the reciprocal (dual) basis 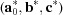 of the basis 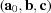. The (centered) lattice bases of the *P*2_1_/*c* and *I*2/*a* polytypes are 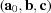 and 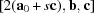, respectively (for 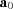 and *s* see §3.2). These correspond to the reciprocal lattices 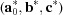 and 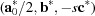. Supposing 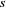 = −¼ and neglecting the minor lattice desymmetrization, the reciprocal bases of both polytypes are related by 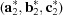 = 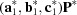 with 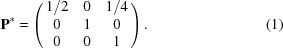
It has to be noted that the lattices of both polytypes are related by a shear mapping and therefore 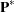 does not represent a classical twin operation. Owing to the centering of the *I*2/*a* polytype, reflections of both polytypes overlap perfectly only on rods 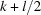 = 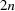, 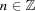 (Fig. 10*a*) ▸. These overlapping reflections are located on a *C*-centered lattice and correspond to the family structure. They are therefore called *family reflections*. It is easily shown that however long the repetition period or how disordered the stacking, intensity on rods 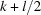 = 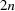, 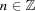, stems only from the family structure and all polytypes contribute equally (proportional to their volume fraction) to these reflections. Note that this is only the case here because adjacent layers are translationally equivalent. Since the Re position in all polytypes is virtually identical to the family structure (§3.5), the family reflections are significantly stronger.

The reflections on the remaining rods (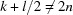) are called characteristic reflections, because they differ among distinct polytypes. The lack of diffuse scattering of these rods indicates that the domains are rather ordered in the crystal under investigation.

## 4. Conclusion   

Crystals of (1) are a further addition to the growing body of structurally characterized allotwins. Clearly, the phenomenon is general and deserves attention. As we have shown here, in principle these problems can be handled with the software packages that are available today. Nevertheless, the lack of seamless integration makes such data manipulations and refinements unnecessarily non-routine. For example, data reduction in the case of the title crystal had to be performed in the triclinic crystal system. Besides being additional work, it is preferred to avoid the thus necessary cell transformations owing to imprecise estimation of standard uncertainties on cell parameters.

Moreover, the files for the deposition of structural data have to be significantly manually edited. Great care is needed to avoid introduction of errors, which will not be caught by the usual automated checks. In particular, some of the statistical concepts seem to be ill-defined. It is, for example, not immediately obvious what an independent observation is in the case of a pair of reflections that was determined as overlapping in one but non-overlapping in a different scan.

We conclude that more work, from theoreticians as well as software vendors, is needed to bring the software-assisted characterization of such crystals to the level it deserves.

## Supplementary Material

Crystal structure: contains datablock(s) RePNP1, RePNP2. DOI: 10.1107/S205252061701006X/ps5063sup1.cif

Structure factors: contains datablock(s) I. DOI: 10.1107/S205252061701006X/ps5063RePNP1sup2.hkl

Structure factors: contains datablock(s) I. DOI: 10.1107/S205252061701006X/ps5063RePNP2sup3.hkl

CCDC references: 1560699, 1560700

## Figures and Tables

**Table 1:** Experimental details

Crystal data	
*T* (K)	200
θ range (°)	1.70–30.15
Radiation	Mo 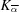
Crystal description, color	Block, yellow
Crystal size (mm)	0.25 × 0.35 × 0.45

Data collection	
Diffractometer	Bruker KAPPA APEX II CCD
Absorption correction	Multi-scan, *TWINABS*
*T*_min_, *T*_max_	0.10–0.27
No. of measured reflections	33905
No. of independent reflections	12472
No. of observed reflections (*I* ≥ 3σ*I*)	7417
*R*_int_	0.0385

Refinement	
*R*[*F* ^2^ > 3σ(*F* ^2^)], *wR*(*F*), *S*	0.0476, 0.0526, 1.71
No. of parameters, restraints	452, 0

**Table 2:** Structural data of both polytypes of complex (1)

	*P*2_1_/*c* polytype	*I*2/*a* polytype
Crystal data		
Chemical formula	C_21_H_35_ClNO_2_P_2_Re	C_21_H_35_ClNO_2_P_2_Re
*M*_r_	617.12	617.12
Crystal system, space group	Monoclinic, *P*2_1_/*c*	Monoclinic, *I*2/*a*
*a*, *b*, *c* (Å)	9.6475 (7), 10.7392 (8), 25.629 (2)	18.6854 (13), 10.7708 (8), 25.599 (3)
β (°)	68.684 (3)	107.480 (4)
*V* (Å^3^)	2473.7 (3)	4914.1 (7)
*Z*, *Z*′	4, 1	8, 1

Refinement		
Δρ_max_, Δρ_min_ (e Å^−3^)†	−2.28, 3.48	−1.61, 2.22
Volume fraction (%)	20.66 (10)	79.34 (10)

† *F*_obs_ attributed to the domains according to the *F*_calc_ ratios.

**Table 3:** Operations relating adjacent 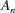 layers in the *P*2_1_/*c* and *I*2/*a* polytypes of (1) Glide reflections and (screw) rotations are in the [010] direction. Symbol subscripts using the generalizations of Dornberger-Schiff & Grell-Niemann (1961 ▸) are given with respect to the basis 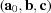.

*P*2_1_/*c*		*I*2/*a*	
Operation	Intrinsic translation	Operation	Intrinsic translation
2_1_	**b**/2	2	–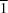	–	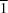	–
*t*	**a**_0_	*t*	**a**_0_ + **b**/2 + *s* **c**
*n*_1,2_	**a**_0_ + **c**/2	*n*_2s,2_	**a**_0_ + *s* **c**

**Table 4:** Distances *d* between corresponding non-H atoms of the polytypes of (1)

Atom	*d* (Å)	Atom	*d* (Å)
Re1	0.277	C8	0.376
Cl1	0.129	C9	0.436
P1	0.309	C10	0.430
P2	0.255	C11	0.242
O1	0.516	C12	0.212
O2	0.361	C13	0.224
N1	0.296	C14	0.364
C1	0.293	C15	0.402
C2	0.279	C16	0.425
C3	0.294	C17	0.262
C4	0.208	C18	0.229
C5	0.225	C19	0.281
C6	0.354	C20	0.395
C7	0.170	C21	0.446
